# Association of Homologous and Heterologous Vaccine Boosters With SARS-CoV-2 Infection in BBIBP-CorV Vaccinated Healthcare Personnel

**DOI:** 10.7759/cureus.27323

**Published:** 2022-07-27

**Authors:** Sungsoo Park, Katrine K Gatchalian, Hyeyoung Oh

**Affiliations:** 1 Department of Pulmonology, Sheikh Khalifa Specialty Hospital, Ras Al Khaimah, ARE; 2 Department of Pulmonary and Critical Care Medicine, Seoul National University Hospital, Seoul, KOR; 3 Environmental Safety Healthcare Provider Team, Sheikh Khalifa Specialty Hospital, Ras Al Khaimah, ARE; 4 Department of Family Medicine, Sheikh Khalifa Specialty Hospital, Ras Al Khaimah, ARE; 5 Department of Family Medicine, Seoul National University Hospital, Seoul, KOR

**Keywords:** healthcare personnel, covid-19 vaccine booster shot, covid-19 vaccines, sars-cov-2, covid-19

## Abstract

Vaccines against severe acute respiratory syndrome coronavirus 2 (SARS-CoV-2) have played a crucial role in mitigating the coronavirus disease 2019 (COVID-19) pandemic. However, few studies have addressed the optimal booster vaccine type in recipients of the primary series of BBIBP-CorV (an inactivated virus vaccine developed by Sinopharm). This study aimed to estimate the association between the heterologous or homologous COVID-19 vaccination and SARS-CoV-2 infection. The study enrolled healthcare personnel (HCP) who had completed two doses of BBIBP-CorV between November 2020 and September 2021. The associations between SARS-CoV-2 infection and boosters were measured using multivariable logistic regression, comparing the odds of a positive COVID-19 test result between the no booster group and booster groups (BNT162b2 {Pfizer-BioNTech COVID-19 vaccine} group and BBIBP-CorV group, respectively). A total of 495 HCP comprising 326 (65.9%) in the BNT162b2 group, 121 (24.4%) in the no booster group, and 48 (9.7%) in the BBIBP-CorV group enrolled. One hundred thirty-six cases (27.5%) tested positive for COVID-19. The odds ratios for testing positive after booster dose were 0.401 (95% CI: 0.187-0.860, p = 0.019) and 0.446 (95% CI: 0.170-1.167, p = 0.100) for BNT162b2 and BBIBP-CorV group, respectively. The BNT162b2 booster in HCP after a second dose of BBIBP-CorV, relative to no booster, and the BBIBP-CorV booster, was associated with protection against laboratory-confirmed COVID-19.

## Introduction

Since the emergence of severe acute respiratory syndrome coronavirus 2 (SARS-CoV-2) in December 2019, the World Health Organization (WHO) has granted emergency use listing to 10 coronavirus disease 2019 (COVID-19) vaccines as of 02 March 2022 [1]. These vaccines against SARS-CoV-2 have played a crucial role in mitigating the COVID-19 pandemic. However, the effectiveness of messenger RNA (mRNA) and viral vector vaccines against COVID-19 decreases over time [2,3]. Additionally, the emergence of SARS-CoV-2 variants raised concerns about the efficacy of the two-dose regimen. The Omicron variant, the current dominant variant since winter of 2021, could escape most neutralizing antibodies [4]. Even though the effectiveness of boosters was less for the Omicron variant than for the Delta variant, boosters were still associated with protection against Omicron. [5]. Thus, many countries implemented the third shot to boost the existing immune response.

BBIBP-CorV, an inactivated vaccine developed by a Chinese state-owned Sinopharm, showed comparable efficacy initially [6] and the least adverse effects [7]. Furthermore, BBIBP-CorV has many advantages with regard to storage, distribution, and stability. Inactivated COVID-19 vaccines do not need an ultracold chain and can be stored at room temperature for a long time [8], enabling many low-income or mid-income counties to achieve a high vaccination rate in a short time. Thus, inactivated COVID-19 vaccines were the most commonly administered globally [9].

Like other vaccines, the immunity of BBIBP-CorV significantly declined 180 days after completing the primary vaccine series [10]. The efficacy of a BBIBP-CorV booster dose after the primary series of BBIBP-CorV (homologous) increased 86% against symptomatic disease and 94% against severe disease. However, the WHO recommended a booster other than BBIVP-CorV following receipt of two doses of BBIBP-CorV (heterologous), based on studies for other inactivated vaccines and limited data from Bahrain [11]. So far, there have been little clinical data on the effect of homologous or heterologous vaccination in recipients of BBIBP-CorV, in contrast to CoronaVac, another inactivated vaccine. If the BBIBP-CorV booster is as effective as the mRNA vaccine booster, it could contribute to achieving a high booster vaccination rate and prevent breakthrough infections.

The United Arab Emirates approved BBIBP-CorV on 9 December 2020 [12]. The Ministry of Health and Prevention granted the emergency registration to Pfizer-BioNTech’s COVID-19 vaccine (BNT162b2) on 22 December 2020. Since only the Dubai Health Authority started vaccination using BNT162b2 in the initial stage, most healthcare personnel (HCP) in our institution received a primary series of BBIBP-CorV followed by a BNT162b2 booster dose. All HCP were obliged to get a COVID-19 vaccine from 20 June 2021. COVID-19 cases surged from December 2021 to February 2022 [13], and most HCP got tested during this period, enabling the researchers to evaluate the effectiveness of boosters.

We hypothesized that heterologous vaccination in recipients of BBIBP-CorV was associated with more protection against COVID-19 than homologous vaccination, based on previous studies using BBIBP-CorV and CoronaVac [14-17]. This study aimed to estimate the association between the heterologous or homologous COVID-19 vaccination and SARS-CoV-2 infection during the Omicron-predominant period.

## Materials and methods

Study population and data collection

All HCP aged 18 years or older who had completed two doses of the BBIBP-CorV vaccine between November 2020 and September 2021 (n=599) were eligible from a database search in the hospital. To enter the study population, we required one SARS-CoV-2 polymerase chain reaction (PCR) test at least 14 days after the second dose. Subjects who did not perform PCR tests from December 2021 to February 2022 or received boosters other than BNT162b2 or BBIBP-CorV vaccine were excluded. Boosters were not expected to induce an immunologic response for the first seven days following the third dose. 

Demographic information and COVID-19 vaccination records were collected, including vaccine type and date. Demographic information included age, sex, staff role, and nationality. Information regarding vaccine type and the date was confirmed by source verification (e.g., Al Hosn app, vaccine registries, and vaccination card). All SARS-CoV-2 PCR tests conducted after two doses of BBIBP-CorV until 24 February 2022 were obtained.

All patients in our institution were admitted to negative pressure rooms after a routine screening PCR test for SARS-CoV-2 from 13 April 2020 onwards. As soon as the PCR result was positive, the patient was transferred to the designated COVID-19 hospital. As a result, workplace exposure to HCP was minimized. 

SARS-CoV-2 testing and reporting

The presence of SARS-CoV-2 in a nasopharyngeal swab was confirmed by the Allplex2019-nCoV assay reverse transcription PCR kit (Seegene, Seoul, Korea), targeting the envelope (E), RNA-dependent RNA polymerase (RdRp) (Spike (S) gene co-detected in the upgraded PCR kit version), and nucleocapsid (N) genes of SARS-CoV-2. The sample was reported positive when three target signals were detected at cycle threshold <40, according to the manufacturer’s interpretative criteria. SARS-CoV-2 infection was defined as a positive PCR test, regardless of symptoms or the reason for testing. A positive PCR result at least three months before a new positive PCR finding was deemed ‘previous SARS-CoV-2 infection’.

Statistical analysis

The associations between SARS-CoV-2 infection and boosters were measured using multivariable logistic regression. We compared the odds of a positive PCR test result among HCP who had received the booster dose more than seven days previously (BNT162b2 group and BBIBP-CorV group, respectively) with that among HCP without a booster dose (no booster group). The final model included age, gender, staff role (physician, nurse, allied health professional, or admin), nationality (the Middle East and North Africa, Filipino, Korean, South Asian, or other), previous SARS-CoV-2 infection, and time from the last dose to COVID-19 test (< six months vs. > six months) to adjust for possible confounders.

Sensitivity analysis was conducted for those other than the BBIBP-CorV group, as HCP who received the BBIBP-CorV booster were small, and the BBIBP-CorV booster doses were given later than the BNT162b2 booster group. To exclude the effect of previous SARS-CoV-2 infection, a test was performed for participants who did not have a history of COVID-19. A two-sided alpha level of 0.05 defined statistical significance. Analyses were conducted using R statistical software, version 0.98.1103 (RStudio, Boston, MA).

## Results

A total of 495 healthcare personnel who completed a primary series of BBIVP-CorV between 25 November 2020, and 21 September 2021, were enrolled (Table 1). Of a total of 599 HCP who were eligible, around 100 HCP who did not perform PCR tests from December 2021 to February 2022 were excluded. The median age of participants was 36 years (IQR; interquartile range 32-42), and 263 HCP (263/495, 53.1%) were female. The staff role and nationality proportions were different among the three groups (p = 0.023 and p <0.001, respectively). Around one-fifth of participants (100/495, 20.2%) had a history of previous infection, with a similar proportion among the three booster groups.

**Table 1:** Characteristics of enrolled healthcare personnel. IQR: Interquartile range; MENA: Middle East and North Africa; BNT162b2: Pfizer-BioNTech COVID-19 vaccine; BBIBP-CorV: An inactivated virus vaccine against COVID-19 developed by Sinopharm

	Total (N=495)	No booster (N=121)	BNT162b (N=326)	BBIBP-CorV (N=48)	p-value
Age, Median (IQR), years	36 (32,42)	35 (32,41)	37 (32,43)	35 (32,40)	0.341
Female	263 (53.1%)	57 (47.1%)	184 (56.4%)	22 (45.8%)	0.121
Staff role					0.023
Physician	75 (15.2%)	25 (20.7%)	44 (13.5%)	6 (12.5%)	
Nurse	218 (44.0%)	41 (33.9%)	161 (49.4%)	16 (33.3%)	
Allied health professional	97 (19.6%)	23 (19.0%)	62 (19.0%)	12 (25.0%)	
Admin	105 (21.2%)	32 (26.4%)	59 (18.1%)	14 (29.2%)	
Nationality					<0.001
MENA	170 (34.3%)	68 (56.2%)	74 (22.7%)	28 (58.3%)	
Filipino	124 (25.1%)	10 (8.3%)	110 (33.7%)	4 (8.3%)	
Korean	90 (18.2%)	9 (7.4%)	78 (23.9%)	3 (6.3%)	
South Asian	94 (19.0%)	30 (24.8%)	53 (16.3%)	11 (22.9%)	
Other	17 (3.4%)	4 (3.3%)	11 (3.4%)	2 (4.2%)	
Previous infection	100 (20.2%)	32 (26.4%)	56 (17.2%)	12 (25.0%)	0.065
Days after Previous infection, Median (IQR)	280 (222, 387)	286 (220, 399)	263 (223, 368)	338 (177, 498)	0.785
2^nd^ dose to booster, Median (IQR), days			193 (165,219)	216 (195, 253)	<0.001
Booster dose to test, Median (IQR), days			117 (79, 153)	102 (55,130)	0.112
2^nd^ dose to test, Median (IQR), days	329 (304,349)	308 (211,336)	334 (315,353)	333 (296,347)	<0.001
Positive test	136 (27.5%)	49 (40.5%)	75 (23.0%)	12 (25.0%)	0.001

Ninety-seven percent of HCP (480/495) received a second dose from January 2021 to June 2021. While 65.9% of HCP (326/495) received a BNT162b2 booster, 24.4% of HCP (121/495) did not receive a booster dose. Only 48 HCP (9.7%) received a BBIBP-CorV booster from June 2021 to January 2022. The BBIBP-CorV group had a longer median interval between the 2nd dose and the booster dose than the BNT162b2 group (216 days vs. 193 days, p <0.001). Median intervals between boosters and COVID-19 tests between the two booster groups were similar (117 days vs. 102 days, p = 0.112). One hundred thirty-six cases (136/495, 27.5%) tested positive for COVID-19, with the highest infection rate in the no booster group (40.5%). 

The odds ratios for testing positive of each booster were 0.401 (95% CI: 0.187-0.860, p = 0.019) and 0.446 (95% CI: 0.170-1.167, p = 0.100) for BNT162b2 and BBIBP-CorV group, respectively (Table 2). The odds ratios of previous infection and interval at six months or less after the last dose were 0.475 and 1.052 (95% CI: 0.269-0.837, p = 0.010; 95% CI: 0.516-2.143, p = 0.889, respectively). Repeating the analysis for those other than the BBIBP-CorV group confirmed the study results. After restricting the analysis only to HCP without a history of COVID-19, the BNT162b2 booster was marginally associated with a positive COVID-19 test (odds ratio 0.424, 95% CI: 0.179-1.003, p = 0.051).

**Table 2:** Odds ratios for SARS-CoV-2 infection. CI; confidence interval, SARS-CoV-2; severe acute respiratory syndrome coronavirus 2; BNT162b2: Pfizer-BioNTech COVID-19 vaccine; BBIBP-CorV: Sinopharm-Beijing Institute of Biological Products COVID-19 vaccine *The analysis was adjusted for age, gender, nationality, and staff role.

	Odds ratio (95% CI)	p-value
BNT162b2 booster	0.401 (0.187, 0.860)	0.019
BBIBP-CorV booster	0.446 (0.170, 1.167)	0.100
Previous SARS-CoV-2 infection	0.475 (0.269, 0.837)	0.010
<6 months from the last dose to test	1.052 (0.516, 2.143)	0.889

## Discussion

The researchers compared the association between BNT162b2 and BBIBP-CorV booster doses and SARS-CoV-2 infection among HCP in the Northern Emirates. The study showed that the BNT162b2 booster in recipients of BBIBP-CorV had a preventive effect on COVID-19. The effectiveness of a third BBIBP-CorV booster following the two-dose BBIBP-CorV regimen was not comparable with the BNT162b2 booster.

A previous study showed a better immunologic response to the mRNA booster dose after the primary series of BBIBP-CorV compared with the BBIBP-CorV booster [14]. The immune response was more robust in heterologous boosting than homologous boosting after two doses of CoronaVac, another inactivated COVID-19 vaccine [15]. A BNT162b2 booster dose after two doses of CoronaVac proved its effectiveness against SARS-CoV-2 infection in Brazil [16]. Heterologous boosters with AZD1222 (Oxford-AstraZeneca COVID-19 vaccine) or BNT162b2 were superior to homologous boosters with CoronaVac for all outcomes [17]. Similarly, the current studies indicate that a heterologous booster after a primary series of BBIBP-CorV is associated with fewer SARS-CoV-2 infections.

Likewise, we cannot find an association between SARS-CoV-2 infection and the BBIBP-CorV booster. There may be several explanations. First, the number of BBIBP-CorV group members was small, and the BBIBP-CorV group had more staff who had previously been infected with SARS-CoV-2. Thus, the associations may be underestimated. Second, the Omicron variant has caused a surge of COVID-19 cases globally since winter 2021 [18]. The Omicron variant showed lower neutralizing sensitivity than other variants after a BBIBP-CorV homologous booster vaccination [19]. This distinctive aspect of Omicron may attenuate the protective effect of BBIBP-CorV more than the mRNA vaccine. Compared to two or three doses of the homologous inactivated vaccine, heterologous vaccination enhanced the immune response against the Omicron variant [20]. 

A previous infection was associated with protection against reinfection despite the median interval between the previous infection and PCR testing being almost nine months. Recent studies demonstrated that the risk of reinfection was low for up to 20 months [21]. The duration of immunity after infection is not consistent between individuals due to various host factors. Participants in the present study were young and did not have significant comorbidities to compromise immune status. These characteristics may contribute to the protective effect of the previous infection for up to nine months.

The strength of this study is that participants were HCP, a homogenous population in a uniform hospital setting, which may exclude other confounders, such as old age and immunosuppressive state. In addition, we gathered data at a median of three-four months after boosters before immunity by booster dose starts to decline significantly, making the result robust. The protection by mRNA vaccines booster shots against emergent or urgent care encounters waned over four months more during the Omicron surge [22].

However, the current study has some limitations. It is a single-center study not dealing with safety and adverse effect. As a result of the difficulty in getting information from the general population, participants are only young HCP, limiting the results' generalizability. Significant comorbidities were not systemically assessed. Representative data to estimate the true prevalence of the Omicron variant was not available. No event of hospitalization or admission to the intensive care unit was reported, so we should be cautious about these outcomes. However, other large-scale studies indicated that inactivated vaccine boosters had a persistent preventive effect on severe COVID-19 outcomes [15]. The goal of COVID-19 vaccination is shifting from the prevention of COVID-19 to the prevention of severe disease and death during the Omicron-predominant period. Thus, inactivated vaccine boosters may still be essential tools to tackle COVID-19, especially if the heterologous booster is contraindicated or not available.

## Conclusions

A BNT162b2 booster in HCP after a second dose of BBIBP-CorV, relative to no booster and BBIBP-CorV booster, was associated with protection against laboratory-confirmed COVID-19. It can serve as evidence to demonstrate the need for a heterologous booster shot for the public after two doses of BBIBP-CorV. Further study is needed to validate our findings on the BBIBP-CorV booster in general population. 
